# Rcorrector: efficient and accurate error correction for Illumina RNA-seq reads

**DOI:** 10.1186/s13742-015-0089-y

**Published:** 2015-10-19

**Authors:** Li Song, Liliana Florea

**Affiliations:** 1 Department of Computer Science, Johns Hopkins University, Baltimore, 21218 USA; 2 McKusick-Nathans Institute of Genetic Medicine, Johns Hopkins University School of Medicine, Baltimore, 21205 USA

**Keywords:** Next-generation sequencing, RNA-seq, Error correction, *k*-mers

## Abstract

**Background:**

Next-generation sequencing of cellular RNA (RNA-seq) is rapidly becoming the cornerstone of transcriptomic analysis. However, sequencing errors in the already short RNA-seq reads complicate bioinformatics analyses, in particular alignment and assembly. Error correction methods have been highly effective for whole-genome sequencing (WGS) reads, but are unsuitable for RNA-seq reads, owing to the variation in gene expression levels and alternative splicing.

**Findings:**

We developed a *k*-mer based method, Rcorrector, to correct random sequencing errors in Illumina RNA-seq reads. Rcorrector uses a De Bruijn graph to compactly represent all trusted *k*-mers in the input reads. Unlike WGS read correctors, which use a global threshold to determine trusted *k*-mers, Rcorrector computes a local threshold at every position in a read.

**Conclusions:**

Rcorrector has an accuracy higher than or comparable to existing methods, including the only other method (SEECER) designed for RNA-seq reads, and is more time and memory efficient. With a 5 GB memory footprint for 100 million reads, it can be run on virtually any desktop or server. The software is available free of charge under the GNU General Public License from https://github.com/mourisl/Rcorrector/.

**Electronic supplementary material:**

The online version of this article (doi:10.1186/s13742-015-0089-y) contains supplementary material, which is available to authorized users.

## Introduction

Next-generation sequencing of cellular RNA (RNA-seq) has become the foundation of virtually every transcriptomic analysis. The large number of reads generated from a single sample allow researchers to study the genes being expressed and estimate their expression levels, and to discover alternative splicing and other sequence variations. However, biases and errors introduced at various stages during the experiment, in particular sequencing errors, can have a significant impact on bioinformatics analyses.

Systematic error correction of whole-genome sequencing (WGS) reads was proven to increase the quality of alignment and assembly [1–3], two critical steps in analyzing next-generation sequencing data. There are currently several error correction methods for WGS reads, classified into three categories [4]. *K*-spectrum based methods, which are the most popular of the three, classify a *k*-mer as trusted or untrusted depending on whether the number of occurrences in the input reads exceeds a given threshold. Then, for each read, low-frequency (untrusted) *k*-mers are converted into high-frequency (trusted) ones. Candidate *k*-mers are stored in a data structure such as a Hamming graph, which connects *k*-mers within a fixed distance, or a Bloom filter. Methods in this category include Quake [5], Hammer [6], Musket [7], Bless [1], BFC [2], and Lighter [3]. Suffix tree and suffix array based methods build a data structure from the input reads, and replace a substring in a read if its number of occurrences falls below that expected given a probabilistic model. These methods, which include Shrec [8], Hybrid-Shrec [9] and HiTEC [10], can handle multiple *k*-mer sizes. Lastly, multiple sequence alignment (MSA) based methods such as Coral [11] and SEECER [12] cluster reads that share *k*-mers to create a local vicinity and a multiple alignment, and use the consensus sequence as a guide to correct the reads.

RNA-seq sequence data differ from WGS data in several critical ways. First, while read coverage in WGS data is largely uniform across the genome, genes and transcripts in an RNA-seq experiment have different expression levels. Consequently, even low-frequency *k*-mers may be correct, belonging to a homolog or a splice isoform. Second, alternative splicing events can create multiple correct *k*-mers at the event boundaries, a phenomenon that occurs only at repeat regions for WGS reads. In both of these cases, the reads would be erroneously converted by a WGS correction method. Hence, error correctors for WGS reads are generally not well suited for RNA-seq sequences [13].

There is so far only one other tool designed specifically for RNA-seq error correction, called SEECER [12], based on the MSA approach. Given a read, SEECER attempts to determine its context (overlapping reads from the same transcript), characterized by a hidden Markov model, and to use this to identify and correct errors. One significant drawback, however, is the large amount of memory needed to index the reads. Herein we propose a novel *k*-spectrum based method, Rcorrector (RNA-seq error CORRECTOR), for RNA-seq data. Rcorrector uses a flexible *k*-mer count threshold, computing a different threshold for a *k*-mer within each read, to account for different transcript and gene expression levels. It also allows for multiple *k*-mer choices at any position in the read. Rcorrector only stores *k*-mers that appear more than once in the read set, which makes it scalable with large datasets. Accurate and efficient, Rcorrector is uniquely suited to datasets from species with large and complex genomes and transcriptomes, such as human, without requiring significant hardware resources. Rcorrector can also be applied to other types of data with non-uniform coverage such as single-cell sequencing, as we will show later. In the following sections we present the algorithm, first, followed by an evaluation of this and other methods on both simulated and real data. In particular, we illustrate and compare the impact of several error correctors for two popular bioinformatics applications, namely, alignment and assembly of reads.

## Algorithm

### De Bruijn graph

In a first preprocessing stage, Rcorrector builds a De Bruijn graph of all *k*-mers that appear more than once in the input reads, together with their counts. To do so, Rcorrector uses Jellyfish2 [14] to build a Bloom counter that detects *k*-mers occurring multiple times, and then stores these in a hash table. Intuitively, the graph encodes all transcripts (full or partial) that can be assembled from the input reads. At run time, for each read the algorithm finds the closest path in the graph, corresponding to its transcript of origin, which it then uses to correct the read.

### Read error correction: the path search algorithm

As with any *k*-spectrum method, Rcorrector distinguishes among solid and non-solid *k*-mers as the basis for its correction algorithm. A solid *k*-mer is one that passes a given count threshold and therefore can be trusted to be correct. Rcorrector uses a flexible threshold for solid *k*-mers, which is calculated for each *k*-mer within each read sequence. At run time, Rcorrector scans the read sequence and, at each position, decides whether the next *k*-mer and each of its alternatives are solid and therefore represent valid continuations of the path. The path with the smallest number of differences from the read sequence, representing the likely transcript of origin, is then used to correct *k*-mers in the original read.

**Fig. 1:**
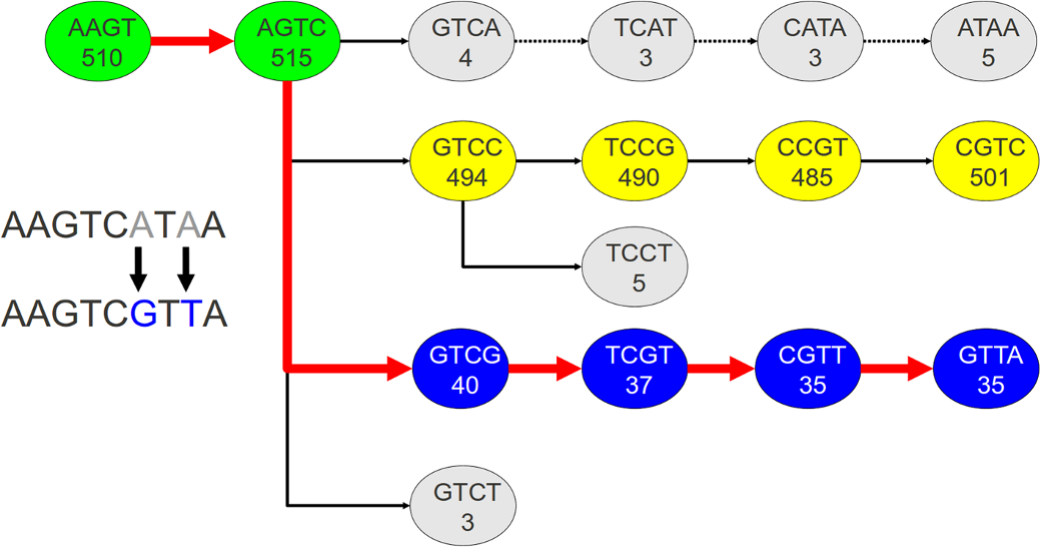
Path extension in Rcorrector. Four possible path continuations at the AGTC *k*-mer (*k*=4) in the De Bruijn graph for the *r*= AAGTCATAA read sequence. Numbers in the vertices represent *k*-mer counts. The first (*top*) path corresponds to the original read’s representation in the De Bruijn graph. The extension is pruned after the first step, AGTC →GTCA, as the count *M*(*GTCA*)=4 falls below the local cutoff (determined based on the maximum *k*-mer count (494) of the four possible successors of AGTC). The second path (*yellow*) has higher *k*-mer counts but it introduces four corrections, changing the read into AAGTCCGTC. The third path (*blue*) introduces only two corrections, to change the sequence into AAGTCGTTA, and is therefore chosen to correct the read. The fourth (*bottom*) path is pruned as the *k*-mer count for GTCT does not pass the threshold. Paths 2 and 3 are likely to indicate paralogs and/or splice variants of this gene

More formally, let *u* be a *k*-mer in read *r* and *S*(*u*,*c*) denote the successor *k*-mer for *u* when appending nucleotide *c*, with *c*∈{A,C,G,T}. For example, in Fig. 1, *S*(AAGT,C)=AGTC, *k*=4. Let *M*(*u*) denote the multiplicity of *k*-mer *u*. To find a start node in the graph from which to search for a valid path, Rcorrector scans the read to identify a stretch of two or more consecutive solid *k*-mers, and marks these bases as solid. Starting from the longest stretch of solid bases, it proceeds in both directions, one base at a time as described below. By symmetry, we only illustrate the search in the 5 ^′^→3^′^ direction.

Suppose *u*=*r*_*i*_*r*_*i*+1_…*r*_*i*+*k*−1_ is the *k*-mer starting at position *i* in read *r*. Rcorrector considers all possible successors *S*(*u*,*c*), *c*∈{A,C,G,T}, and their multiplicities *M*(*S*(*u*,*c*)) and determines which ones are solid based on a locally defined threshold (see below). Rcorrector tests all the possible nucleotides for position *i*+*k* and retains those that lead to solid *k*-mers, and then follows the paths in the De Bruijn graph from these *k*-mers. Multiple *k*-mer choices are considered in order to allow for splice variants. If the nucleotide in the current path is different from *r*_*i*+*k*_, then it is marked as a correction. When the number of corrections in the path exceeds an *a priori* defined threshold, Rcorrector terminates the current search path and starts a new one. In the end, Rcorrector selects the path with the minimum number of changes and uses the path’s sequence to correct the read. To improve speed, Rcorrector does not attempt to correct solid positions, and gradually decreases the allowable number of corrections if the number of searched paths becomes large.

### A flexible local threshold for solid *k*-mers

Let *u* be the *k*-mer starting at position *i* in the read, as before. Unlike with WGS reads, even if the multiplicity *M*(*S*(*u*,*r*_*i*+*k*_)) of its successor *k*-mer is very low, the base *r*_*i*+*k*_ may still be correct, for instance sampled from a low-expression transcript. Therefore, an RNA-seq read error corrector cannot simply use a global *k*-mer count threshold. Rcorrector uses a locally defined threshold as follows. Let *t*= max*cM*(*S*(*u*,*c*)), calculated over all possible successors of *k*-mer *u* encoded in the De Bruijn graph. Rcorrector defines the local threshold at run time, *f*(*t*,*r*), as the smaller of two values, a *k*-mer-level threshold and a read-level one: *f*(*t*,*r*)= min(*g*(*t*),*h*(*r*)).

The *k*-mer-level threshold is defined as $g(t)=\alpha t + 6\sqrt {\alpha t}$, where *α* is a global variation coefficient. Specifically, *α* is determined for each dataset from a sample of 1 million high-count *k*-mers (multiplicities over 1,000), as follows. Given the four (or fewer) possible continuations of a *k*-mer, Rcorrector calculates a value equal to the ratio between the second highest and the highest multiplicities. Then, *α* is chosen as the smallest such value larger than 95 % of those in the sample. This criterion ensures that only *k*-mers that can be unambiguously distinguished from their alternates will be chosen; lowering this parameter value will reduce the stringency. Note that the *k*-mer-level threshold is the same for a *k*-mer in all read contexts, but differs by *k*-mer.

To calculate the read-level threshold, Rcorrector orders all *k*-mers in the read by decreasing multiplicities. Let *x* be the multiplicity before the first sharp drop (> 2-fold) in this curve. Rcorrector then uses *h*(*r*)=*g*(*x*) as the read-level threshold. Refinements to this step to accommodate additional lower-count paths are described below.

### Refinements

#### Clustered corrections

Once a set of corrections has been determined for a read, Rcorrector scans the read and selectively refines those at nearby positions. The rationale for this step is that the likelihood of two or more clustered errors is very low under the assumed model of random sequencing errors, and the read may instead originate from a paralog. More specifically, let *u*_*i*_ and *u*_*j*_ be the *k*-mers ending at two positions *i* and *j*, with *j*−*i*<*k*, and *M*(*u*_*i*_) and *M*(*u*_*j*_) their multiplicities. To infer the source for the *k*-mer, Rcorrector uses the local read context and tests for the difference in the multiplicities of *k*-mers before correction. If the difference is significant, then it is a strong indication for a cluster of sequencing errors. Otherwise (i.e., if 0.5<*M*(*u*_*i*_)/*M*(*u*_*j*_)<2), then the *k*-mers are likely to have originated from the same path in the graph, corresponding to a low-expression paralog, and the read is deemed to be correct. Rcorrector will revert corrections at positions *i* and *j* and then iteratively revisit all corrections within distance *k* from those previously reverted.

#### Unfixable reads

Rcorrector builds multiple possible paths for a read and in the end chooses the path with the minimum number of base changes. If the number of changes over the entire read or within any window of size *k* exceeds an *a priori* determined threshold, the read is deemed ‘unfixable’. There are two likely explanations for unfixable reads: *i*) the read is correct, and originates from a low-expression transcript for which there is a higher-expression homolog present in the sample; and *ii*) the read contains too many errors to be rescued.

In the first case, Rcorrector never entered the true path in the graph during the extension, and hence the read was incorrectly converted to the high-expression homolog. To alleviate this problem, Rcorrector uses an iterative procedure to lower the read-level threshold *h*(*r*) and allow lower count *k*-mers in the path.

Specifically, Rcorrector looks for the next sharp drop in the *k*-mer multiplicity plot to define a new and reduced *h*(*r*), until there is no such drop or the number of corrections is within the set limits.

#### PolyA tail reads

The presence of polyA tail sequences in the sample will lead to *k*-mers with mostly A or T bases. Because their multiplicities are derived from a mixture distribution from a large number of transcripts, these *k*-mers are ignored during the correction process. Rcorrector will consequently not attempt to correct such *k*-mers.

#### Paired-end reads

With paired-end reads, Rcorrector leverages the *k*-mer count information across the two reads to improve the correction accuracy. In particular, it chooses the smaller of the two read-level thresholds as the common threshold for the two reads. In doing so, it models the scenario where the fragment comes from a low-expression isoform of the gene, with one of the reads specific to this isoform and the other shared among multiple, higher-expression isoforms. In this case, the lower of the two read-level thresholds better represents the originating transcript.

## Findings

We evaluate Rcorrector for its ability to correct Illumina sequencing reads, both simulated and real. We include in the evaluation four other error correctors: SEECER (v0.1.3), which is the only other tool specifically designed for RNA-seq reads, as well as at least one representative method for each of the three classes of WGS error correction methods. These include Musket (v1.1) and BFC (r181) for *k*-spectrum, Hybrid-Shrec (Hshrec) for suffix tree and suffix array, and Coral (v1.4) for MSA-based methods. Since many tools are sensitive to the *k*-mer size *k*, we test different *k*-mer sizes for each tool where applicable and report the result that produces the best performance. We assess the impact of all programs on two representative bioinformatics applications, read alignment and read assembly. Lastly, we show that Rcorrector can be successfully applied to other types of data exhibiting non-uniform read coverage, such as single-cell sequencing reads.

### Evaluation on simulated data

In a first test, we evaluated all programs on a simulated dataset containing 100 million 100 bp long paired-end reads. Reads were generated with FluxSimulator [15] starting from the human GENCODE v.17 gene annotations. Errors were subsequently introduced with Mason [16]; error rates were extracted from alignments of same-length Illumina Human Body Map reads (Additional file 1, Section S1). As in [4], we evaluate the accuracy of error corrections by inspecting how each base was corrected. Let true positives (TP) be the number of error bases that are converted into the correct nucleotide; false positives (FP) the number of error-free bases that are falsely corrected; and false negatives (FN) the number of error bases that are not converted or where the converted base is still an error. We use the standard measures of *Recall*=*TP*/(*TP*+*FN*), *Precision*=*TP*/(*TP*+*FP*), and *F*_*score*=2∗*Recall*∗*Precision*/(*Recall*+*Precision*) to evaluate all methods. For each tool we test different *k*-mer sizes and report the result with the best *F*_*score*.

Accuracy values and performance measurements for the six error correctors are shown in Table 1. All programs were run on a 256 GB RAM machine with a 48-core 2.1 GHz AMD Opteron(TM) processor, with 8 threads. Here and throughout the manuscript, all measures are expressed in percentages. The overall sensitivity is below 90 % for all methods due to the large number of polyA reads generated by FluxSimulator, which are left unchanged. Rcorrector has the best overall performance by all measures, with 88 % sensitivity and greater than 99 % precision, followed closely by SEECER. Rcorrector is also virtually tied with BFC for the fastest method, and is among the most memory efficient. In particular, at 5 GB RAM for analyzing 100 million reads, it required 12 times less memory than SEECER and can easily fit in the memory of most desktop computers (Table 1).

**Table 1:** Accuracy of the six error correction methods on the 100 million simulated reads

Program	*k*	Recall	Precision	F-score	Run time	Memory
					(min)	(GB)
SEECER	31	87.13	96.93	91.77	177	61
HShrec	-	69.53	31.74	43.58	13641	30
Coral	31	58.35	85.14	69.25	1391	81
Musket	27	78.24	96.90	86.58	152	*4*
BFC	27	80.45	97.91	88.32	*111*	6
Rcorrector	27	*88.94*	*99.84*	*94.07*	118	5

Best performers in each category are highlighted in italic. All programs were run multithreaded, with eight threads

The difficulty of error correction is expected to vary with the expression level of transcripts. Correcting reads from low-expression transcripts is particularly challenging because the error-containing *k*-mers cannot be easily distinguished on the basis of frequency. To assess the performance of the various tools with transcript expression levels, we divide the simulated transcripts into low-, medium-, and high-expression groups based on their relative abundance *A* assigned by FluxSimulator (low, *A*<5*e*^−7^; medium, 5*e*^−7^<*A*<0.0001; and high, *A*>0.0001). The results of each tool on the three subclasses are shown in Table 2. Most tools perform well on the high-expression dataset, with the exception of Coral (low sensitivity) and Hshrec (low precision). However, the performance for all methods, especially sensitivity, drops for reads from low-expression transcripts. Rcorrector has the best or comparable sensitivity and precision for each of the three classes of transcripts. Both Rcorrector and SEECER are significantly more precise (>86 % in all categories) and more sensitive than methods designed for DNA reads, especially for reads from low-expression transcripts.

**Table 2:** Accuracy of six error correction methods on 100 million simulated reads, by expression level of transcripts. *k*-mer sizes used are those in Table 1

Program	Recall	Precision	F-score
Low expression			
SEECER	32.78	*90.54*	48.14
HShrec	24.77	0.81	1.56
Coral	31.88	64.60	42.69
Musket	13.88	33.94	19.71
BFC	25.18	58.37	35.19
Rcorrector	*39.40*	86.62	*54.16*
Medium expression			
SEECER	86.58	97.05	91.51
HShrec	70.57	19.57	30.64
Coral	89.07	85.12	87.05
Musket	72.02	92.16	80.86
BFC	*89.12*	96.88	92.84
Rcorrector	87.73	*99.66*	*93.31*
High expression			
SEECER	87.39	96.90	91.90
HShrec	69.22	41.67	52.02
Coral	47.59	85.17	61.06
Musket	80.50	98.53	88.61
BFC	77.47	98.35	86.67
Rcorrector	*89.42*	*99.91*	*94.37*

Best performers are highlighted in italic

### Real datasets

For a more realistic assessment, we applied the tools to three real datasets that vary in their sequencing depth, read length, amount of sequence variation, and application area (Table 3 and Additional file 1: Section S2). These include a plant RNA-seq dataset (peach embryos and cotyledons; SRA accession SRR531865), a lung cancer cell line (SRA accession SRR1062943), and a lymphoblastoid cell line sequenced as part of the GEUVADIS population variation project (SRA accession ERR188021). We use these three sets to evaluate the performance of programs on real data, as well as to illustrate the effects of error correction on the alignment and assembly of RNA-seq reads. Summary statistics for all datasets are shown in Table 3.

**Table 3:** Summary of datasets included in the evaluation

Name	Reads	Read length	Aligned	Perfectly
		(bp)		aligned
Simulated	99,338,716	100	81,994,413	21,070,024
Peach	38,883,238	75	24,775,386	5,617,514
Lung	113,313,254	50	110,771,941	85,160,322
Geuvadis	65,015,656	75	59,130,806	26,468,128

Best performers are highlighted in italic

Unlike for simulated data, the ground truth for each base is unknown, making it impossible to judge performance directly and in an unbiased way. Instead, we use alignment rates to estimate the accuracy of error correction. We tested different *k*-mer sizes for each tool, and chose the one maximizing the total number of matching bases. Statistics for alignments generated with Tophat2 (v2.0.13) [17] are summarized in Table 4. Lacking a true measure of sensitivity, the number and percentage of aligned reads as well as the per base match rate, as introduced in [3], are used to estimate sensitivity at read and base-level, respectively. The per base match rate is computed as the ratio of the total number of all the matching bases to the total number of aligned reads. Likewise, we introduce an alternate measure of specificity, defined as *TN*/(*TN*+*FP*), based on a high-confidence subset of the original reads (Table 4). We extracted those reads that have perfect alignments on the genome, i.e., that had exact sequence matches and the alignment of reads in a pair was concordant. These reads are expected to be predominantly error-free, therefore the proportion of reads that are not corrected represents a measure of specificity. As a caveat, these measures will falsely include those reads that are incorrectly converted to a paralog and aligned at the wrong location in the genome.

**Table 4:** Tophat2 alignments of simulated and real reads

			Simulated reads		
	*k*	Aligned	Observed	Base	Specificity
			rate	match rate	
Original	-	81,994,413	82.540	99.391	-
SEECER	31	85,374,347	*85.943*	*99.988*	99.619
Hshrec	-	77,488,558	78.004	99.888	97.886
Coral	31	84,662,510	85.226	99.745	99.494
Musket	27	84,892,466	85.458	99.906	99.739
BFC	27	84,844,168	85.409	99.918	99.889
Rcorrector	27	85,033,277	85.599	99.986	*99.970*
Peach					
Original	-	24,775,386	63.717	99.198	-
SEECER	27	29,056,747	74.728	*99.879*	99.199
Hshrec	-	24,496,308	63.000	99.265	96.027
Coral	23	28,974,141	74.516	99.316	99.027
Musket	27	28,345,203	72.898	99.256	99.677
BFC	31	26,553,943	68.291	99.278	*99.777*
Rcorrector	23	30,563,388	*78.603*	99.833	99.628
Lung					
Original	-	110,771,941	97.757	99.717	-
SEECER	23	111,261,651	98.189	*99.855*	98.239
Hshrec	-	102,121,932	90.124	99.781	89.786
Coral	23	111,107,133	98.053	99.809	98.330
Musket	27	110,907,828	97.877	99.781	98.698
BFC	23	111,427,773	*98.336*	99.824	99.359
Rcorrector	23	111,198,587	98.134	99.830	*99.599*
Geuvadis					
Original	-	59,130,806	90.949	99.477	-
SEECER	23	61,514,024	94.614	*99.837*	98.530
Hshrec	23	51,669,686	79.473	99.709	87.924
Coral	23	61,399,007	94.437	99.717	98.049
Musket	23	60,450,316	92.978	99.652	97.900
BFC	23	61,870,897	*95.163*	99.775	98.790
Rcorrector	23	61,641,866	94.811	99.814	*99.227*

Best performers are highlighted in italic

Error correction improves alignment rates by 1–11 %, depending on the dataset (Table 4). Note that alignment rates themselves differ with the amount of sequence variation and quality of the data. Rcorrector, SEECER, and BFC take turns in being the most sensitive across the four datasets. However, only Rcorrector and SEECER are consistently ranked among the top results in each category. Rcorrector has the highest or comparable specificity, greater than 99.2 %, in all cases.

We further assess the impact of error correction on improving *de novo* assembly of RNA-seq reads. We used the transcript assembler Oases [18] to assemble the reads *a priori* corrected with each of the methods. To evaluate the quality of the assembled transcripts, we aligned them to the reference genome with the spliced alignment program ESTmapper/sim4db [19], retaining only the best match for each transcript. We use conventional methods and measures to evaluate the performance in reconstructing full-length transcripts [20]. Specifically, we define a match between a reference annotation transcript and the spliced alignment of an assembled transcript if and only if they have identical intron chains, whereas their endpoints may differ. We used the GENCODE v.17 annotations and the peach gene annotations (v1.1) obtained from the Genome Database for Rosaceae as the gold reference for the real datasets, respectively, and the subset of GENCODE transcripts sampled by FluxSimulator for the simulated data. The results, shown in Table 5, again indicate that SEECER, Rcorrector, and BFC have the most impact on improving the accuracy and quality of the assembled transcripts, and show comparable performance. Results were similar, showing Rcorrector and SEECER predominantly producing the top results, when using an alternative assembler, Trinity [21] (Additional file 1: Section S3). Of note, these measures only capture full transcripts, whereas many of the transcripts in the sample will not have enough reads to be assembled fully.

**Table 5:** Oases assembly of simulated and real reads

Program	Simulated		Peach		Lung		Geuvadis	
	Recall	Precision	Recall	Precision	Recall	Precision	Recall	Precision
Original	30.575	48.862	28.879	*16.410*	4.957	10.475	5.997	16.749
SEECER	36.698	*52.181*	*29.752*	16.116	4.944	10.174	6.162	16.639
Hshrec	23.334	47.417	26.132	13.850	3.608	*11.459*	4.266	19.101
Coral	35.039	51.942	29.784	15.881	4.934	10.174	6.170	16.372
Musket	33.845	47.769	28.760	15.991	4.920	10.577	5.846	16.901
BFC	34.789	50.579	29.633	16.211	*5.018*	10.498	6.166	16.509
Rcorrector	*36.763*	52.144	29.355	15.951	5.012	10.478	*6.222*	16.375

Best performers are highlighted in italic

**Fig. 2:**
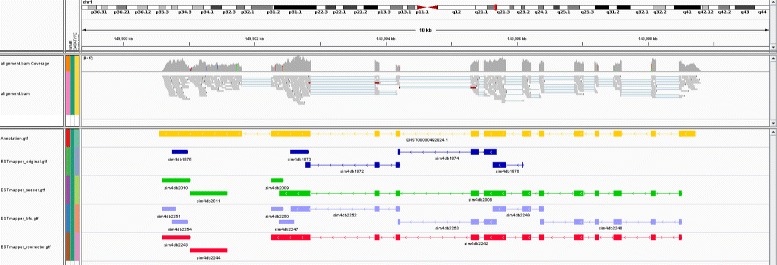
Transcripts assembled from the original and error-corrected reads at the MTMR11 gene locus. Rcorrector (*bottom panel*) improves upon the original reads and leads to the most complete reconstruction of the transcript

Figure 2 illustrates the spliced alignments of a 13 exon transcript at the MTMR11 (myotubularin related protein) gene locus (chr1:149,900,543-149,908,791) assembled with Oases from the simulated reads before and after correction. All methods missed the first intron, which was supported by six error-containing reads, but produced partial reconstructions of the transcript, consisting of multiple contigs (Additional file 1: Section S4). While all error correctors improved upon the original reads, Rcorrector produced the most complete and compact assembly, with only three contigs, including one containing the full reconstruction of exons 1–12.

### Single-cell sequencing

While Rcorrector was designed to correct RNA-seq reads, the method is also applicable to a wider range of problems where read coverage is non-uniform.

Single-cell sequencing has recently emerged as a powerful technique to survey the content and variation within an individual cell. However, PCR amplification of the input DNA introduces biases in read coverage across the genome. We compared Rcorrector with SEECER and the error correction module built into the assembly package SPAdes (3.1.0) [22]. The latter is based on the error corrector BayesHammer [23], which accounts for variable depth coverage. We applied all three methods to correct 29,124,078 *E. coli* K-12 MG1655 Illumina reads [22], then aligned the corrected reads to the *E. coli* K-12 genome with Bowtie2 [24] and assembled them with SPAdes. We evaluated the alignment outcome as described earlier and separately used the package QUAST [25] to assess the quality of the resulting genome assemblies.

As seen in Table 6, Rcorrector results in the largest number of aligned reads, and is also the most specific among the methods. Surprisingly, the built-in SPAdes error corrector shows very low specificity (41.5 %), primarily arising from BayesHammer’s trimming of end sequences for some reads. In contrast, SEECER has very high specificity but relatively low sensitivity, as the number of mapped reads was actually reduced after correction. Rcorrector shows both the highest sensitivity and the highest precision, and is therefore the best choice for this dataset.

**Table 6:** Bowtie2 alignment of single-cell sequencing reads

	*k*	Aligned	Rate	Base match rate	Specificity
Original	-	27,002,682	92.716	98.863	-
SPAdes	-	27,104,190	93.065	99.675	41.482
SEECER	27	26,937,652	92.493	99.507	99.553
Rcorrector	19	27,227,855	*93.489*	*99.711*	*99.998*

Best performers are highlighted in italic

For assembly, both Rcorrector and SEECER lead to longer contigs and better genome coverage compared to the built-in corrector in SPAdes, while Rcorrector additionally produces the smallest number of misassemblies (Table 7). To conclude, Rcorrector can be effectively applied to correct single-cell DNA sequencing reads.

**Table 7:** SPAdes assembly of single-cell sequencing reads. NG50 is the minimum contig length such that the total number of bases in contigs this size or longer represents more than half of the length of the reference genome

	NG50	Misassembly	Edits/100 kbps	Genome
				coverage
Original	105,623	*1*	*6.57*	95.054
SPAdes	109,876	2	7.52	94.903
SEECER	*110,103*	2	7.26	95.059
Rcorrector	*110,103*	*1*	10.02	*95.094*

Best performers are highlighted in italic

## Conclusions

Rcorrector is the first *k*-spectrum based method designed specifically for correcting RNA-seq reads, and addresses several limitations in existing methods. It implements a flexible *k*-mer count threshold, to account for different gene and transcript expression levels, and simultaneously explores multiple correction paths for a read, to accommodate isoforms of a gene. In comparisons with similar tools, Rcorrector showed the highest or near-highest accuracy on all datasets, which varied in their amount of sequencing errors as well as polymorphisms. Also, with a small 5 GB memory footprint for a 100 million read dataset, it required an order of magnitude less memory than SEECER, the only other tool designed specifically for RNA-seq reads. Lastly, Rcorrector was the fastest of all methods tested, taking less than two hours to correct the simulated dataset. Therefore, Rcorrector is an excellent choice for large-scale and affordable transcriptomic studies in both model and non-model organisms.

## Availability and requirements

**Project name:** Rcorrector**Project home page:**http://github.com/mourisl/Rcorrector**Operating system(s):** Unix, Linux**Programming language:** C, C++, Perl**License:** GNU General Public License version 3.0 (GPLv3)**Any restrictions to use by non-academics:** none

## Availability of supporting data

All data sets supporting the analyses are available from the GigaScience GigaDB repository [26].

## Additional file

Additional file 1 **Supplementary material.** Section *S1 -* Command line and error rate parameters for Mason. Section *S2 -* Variation coefficient (*α*) for the four datasets. Section *S3 -* Trinity assembly of simulated and real reads. Section *S4 -* Sim4db spliced alignments of Oases transcripts assembled from original and error-corrected reads. (DOCX 130 kb)
