# Supplementary material for: Rcorrector: efficient and accurate error correction for Illumina RNA-seq reads
Source: Gigascience. 2015 Oct 19;4:48. doi: 10.1186/s13742-015-0089-y (PMC4615873; doi:10.1186/s13742-015-0089-y)
Supplement: Supplementary file 1 — Supplementary material. Section S1 - Command line and error rate parameters for Mason. Section S2 - Variation coefficient (α) for the four datasets. Section S3 - Trinity assembly of simulated and real reads. Section S4 - Sim4db spliced alignments of Oases transcripts assembled from original and error-corrected reads. (DOCX 130 kb) [file 13742_2015_89_MOESM1_ESM.docx]

SUPPLEMENTAL MATERIAL FOR THE PAPER:

RCORRECTOR: EFFICIENT AND ACCURATE ERROR CORRECTION FOR ILLUMINA RNA-SEQ READS

Li Song, Liliana Florea

S1. Command line parameters for Mason.

S2. Variation coefficient (α) for the 4 data sets.

S3. Trinity assembly of simulated and real reads.

S4. Sim4db spliced alignments of Oases transcript assemblies from original and error-corrected reads.

**S1. Command line parameters for Mason simulations**

Parameter values were estimated from Tophat2 alignments of Illumina Human Body Map reads to the human genome hg19, and were used to simulate errors in the Illumina reads previously generated by FluxSimulator:

$MASON illumina -s 265 \

-i -N 99340000 -f -o corr.M.2x100bp.50M.fastq \

-hs 0 -hi 0 -n 100 -sq \

-pi 0 -pd 0 -pmms 1.0 -pmmf hiseq.hs.100bp.pmmf \

-pmm 0.007 -pmmb 0.013 -pmme 0.063 \

-qmb 35.68 -qme 11.86 -qsdb 6.41 -qsde 14.32 \

-mmqmb 29.41 -mmqme 15.84 -mmqsdb 14.40 -mmqsde 15.52 \

corr.M.2x100bp.50M.fasta 2> mason.log

Where hiseq.hs.100bp.pmmf:

0.013 0.007 0.006 0.0055 0.01 0.003 0.003 0.003 0.003 0.003

0.003 0.003 0.003 0.003 0.003 0.003 0.003 0.003 0.003 0.003

0.003 0.003 0.003 0.003 0.003 0.003 0.004 0.003 0.003 0.003

0.003 0.0035 0.003 0.003 0.003 0.003 0.003 0.003 0.003 0.003

0.0035 0.004 0.004 0.004 0.004 0.004 0.004 0.004 0.004 0.004

0.004 0.004 0.0035 0.004 0.004 0.004 0.0045 0.004 0.004 0.0045

0.004 0.0045 0.0045 0.0055 0.005 0.005 0.006 0.005 0.006 0.0265

0.0065 0.006 0.006 0.0065 0.007 0.006 0.006 0.007 0.007 0.007

0.007 0.007 0.0075 0.008 0.009 0.0155 0.0105 0.01 0.01 0.011

0.012 0.013 0.0135 0.0135 0.015 0.017 0.022 0.03 0.0465 0.0635

**S2. Variation coefficient (α) for the 4 data sets**

For each data set (simulated, peach, Geuvadis and lung), we plot the histogram of variation ratios calculated as follows. The variation ratio for a k-mer is defined as the ratio between the second largest and the largest multiplicities among the four continuation k-mers. Hence, the lower the ratio, the more likely it is that the base change at the last position is a sequencing error. Conversely, values closer to 1 are indicative of polymorphisms, whereas middle values are potentially due to sequence differences between paralogs and/or isoforms of a gene. The histograms and distributions were estimated based on 1 million high-count k-mers. Then, we define **α**   value corresponding to the 5^th^ percentile of the distribution.

**
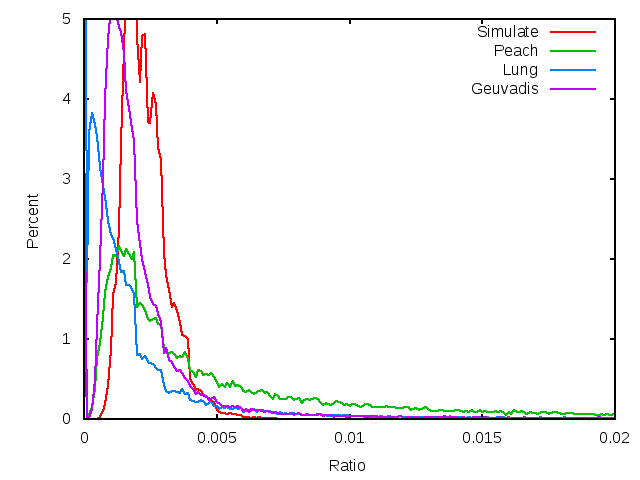
**

**S3. Trinity assembly of simulated and real reads.**

| Program | Simulated | | Peach | | Lung | | Geuvadis | |
| --- | --- | --- | --- | --- | --- | --- | --- | --- |
|  | Recall | Precision | Recall | Precision | Recall | Precision | Recall | Precision |
| Original | 39.245 | 43.042 | 27.593 | 14.688 | 4.109 | 17.960 | 6.049 | 14.994 |
| SEECER | 39.197 | 43.506 | 28.315 | 15.569 | **4.156** | **18.334** | **6.097** | 15.049 |
| Kshrec | 27.600 | 36.859 | 24.432 | **15.860** | 2.875 | 17.404 | 4.151 | 23.136 |
| Coral | 38.612 | 42.518 | 28.252 | 15.682 | 4.113 | 18.132 | 6.044 | 15.050 |
| Musket | 38.507 | 42.134 | 27.537 | 14.684 | 4.099 | 17.909 | 6.045 | 15.023 |
| BFC | 38.410 | 42.724 | 27.886 | 15.194 | 4.123 | 18.173 | 6.061 | **15.093** |
| Rcorrector | **39.459** | **43.843** | **28.545** | 15.709 | 4.117 | 18.052 | 6.081 | 15.026 |

**S4. Sim4db spliced alignments of Oases transcript assemblies from original and error-corrected reads at the MTMR11 gene locus.**

*1. Assemblies of the original reads (5 assemblies)*

*_____________________________________________________*

sim4db1875:sim4begin

1885[252-0-10] 19[149898743-149902995] <242-0-100-complement-unknown>

edef=>Locus_1319_Transcript_1/1_Confidence_1.000_Length_252

ddef=>chr1

1-242 (2001-2242) <242-0-100>

sim4end

sim4db1873:sim4begin

1883[279-0-0] 19[149900550-149904829] <279-0-100-forward-unknown>

edef=>Locus_1317_Transcript_1/1_Confidence_1.000_Length_279

ddef=>chr1

1-279 (2001-2279) <279-0-100>

sim4end

sim4db1872:sim4begin

1882[214-0-0] 19[149900774-149906215] <214-0-100-complement-reverse>

edef=>Locus_1316_Transcript_1/1_Confidence_1.000_Length_214

ddef=>chr1

1-83 (2001-2083) <83-0-100> <-

84-154 (3062-3132) <71-0-100> <-

155-214 (3382-3441) <60-0-100>

sim4end

sim4db1874:sim4begin

1884[357-0-0] 19[149902182-149907694] <357-0-100-complement-reverse>

edef=>Locus_1318_Transcript_1/1_Confidence_1.000_Length_357

ddef=>chr1

1-40 (2001-2040) <40-0-100> <-

41-165 (3117-3241) <125-0-100> <-

166-357 (3321-3512) <192-0-100>

sim4end

sim4db1876:sim4begin

1886[219-0-0] 19[149903632-149907838] <219-0-100-forward-reverse>

edef=>Locus_1320_Transcript_1/1_Confidence_1.000_Length_219

ddef=>chr1

1-203 (2001-2203) <203-0-100> <-

204-219 (2452-2467) <16-0-100>

sim4end

*2. Assemblies of the SEECER-corrector reads (4 assemblies)*

______________________________________________________________

*sim4db2010:sim4begin*

*2017[425-0-0] 19[149898595-149903020] <425-0-100-complement-unknown>*

*edef=>Locus_1344_Transcript_1/1_Confidence_1.000_Length_425*

*ddef=>chr1*

*1-425 (2001-2425) <425-0-100>*

*sim4end*

*sim4db2011:sim4begin*

*2018[571-0-0] 19[149899037-149903588] <569-0-99-complement-unknown>*

*edef=>Locus_1345_Transcript_1/1_Confidence_1.000_Length_571*

*ddef=>chr1*

*1-571 (1981-2551) <569-0-99>*

*sim4end*

*sim4db2009:sim4begin*

*2016[192-0-0] 19[149900256-149904438] <182-0-100-complement-unknown>*

*edef=>Locus_1343_Transcript_1/1_Confidence_1.000_Length_192*

*ddef=>chr1*

*11-192 (2001-2182) <182-0-100>*

*sim4end*

*sim4db2008:sim4begin*

*2015[1737-0-0] 19[149900377-149910518] <1736-0-99-complement-reverse>*

*edef=>Locus_1342_Transcript_1/1_Confidence_1.000_Length_1737*

*ddef=>chr1*

*1-480 (2001-2480) <479-0-99> <-*

*481-551 (3459-3529) <71-0-100> <-*

*552-618 (3779-3845) <67-0-100> <-*

*619-743 (4922-5046) <125-0-100> <-*

*744-1076 (5126-5458) <333-0-100> <-*

*1077-1212 (5707-5842) <136-0-100> <-*

*1213-1291 (5964-6042) <79-0-100> <-*

*1292-1434 (6502-6644) <143-0-100> <-*

*1435-1495 (6815-6875) <61-0-100> <-*

*1496-1617 (7100-7221) <122-0-100> <-*

*1618-1693 (7670-7745) <76-0-100> <-*

*1694-1737 (8098-8141) <44-0-100>*

*sim4end*

*3. Assemblies of the BFC-corrected reads (8 assemblies)*

______________________________________________________________

sim4db2251:sim4begin

2252[207-0-0] 19[149898595-149902802] <207-0-100-complement-unknown>

edef=>Locus_1359_Transcript_1/1_Confidence_1.000_Length_207

ddef=>chr1

1-207 (2001-2207) <207-0-100>

sim4end

sim4db2254:sim4begin

2255[252-0-10] 19[149898743-149902995] <242-0-100-complement-unknown>

edef=>Locus_1362_Transcript_1/1_Confidence_1.000_Length_252

ddef=>chr1

1-242 (2001-2242) <242-0-100>

sim4end

sim4db2250:sim4begin

2251[192-0-0] 19[149900256-149904438] <182-0-100-complement-unknown>

edef=>Locus_1358_Transcript_1/1_Confidence_1.000_Length_192

ddef=>chr1

11-192 (2001-2182) <182-0-100>

sim4end

sim4db2247:sim4begin

2248[231-0-0] 19[149900377-149904608] <231-0-100-forward-unknown>

edef=>Locus_1355_Transcript_1/1_Confidence_1.000_Length_231

ddef=>chr1

1-231 (2001-2231) <231-0-100>

sim4end

sim4db2252:sim4begin

2253[438-0-0] 19[149900550-149906215] <438-0-100-forward-reverse>

edef=>Locus_1360_Transcript_1/1_Confidence_1.000_Length_438

ddef=>chr1

1-307 (2001-2307) <307-0-100> <-

308-378 (3286-3356) <71-0-100> <-

379-438 (3606-3665) <60-0-100>

sim4end

sim4db2253:sim4begin

2254[357-0-0] 19[149902182-149907694] <357-0-100-complement-reverse>

edef=>Locus_1361_Transcript_1/1_Confidence_1.000_Length_357

ddef=>chr1

1-40 (2001-2040) <40-0-100> <-

41-165 (3117-3241) <125-0-100> <-

166-357 (3321-3512) <192-0-100>

sim4end

*sim4db2249:sim4begin*

*2250[411-0-0] 19[149903632-149908412] <411-0-100-forward-reverse>*

*edef=>Locus_1357_Transcript_1/1_Confidence_1.000_Length_411*

*ddef=>chr1*

*1-203 (2001-2203) <203-0-100> <-*

*204-339 (2452-2587) <136-0-100> <-*

*340-411 (2709-2780) <72-0-100>*

*sim4end*

*sim4db2248:sim4begin*

*2249[506-0-0] 19[149904359-149910518] <506-0-100-complement-reverse>*

*edef=>Locus_1356_Transcript_1/1_Confidence_1.000_Length_506*

*ddef=>chr1*

*1-60 (2001-2060) <60-0-100> <-*

*61-203 (2520-2662) <143-0-100> <-*

*204-264 (2833-2893) <61-0-100> <-*

*265-386 (3118-3239) <122-0-100> <-*

*387-462 (3688-3763) <76-0-100> <-*

*463-506 (4116-4159) <44-0-100>*

*sim4end*

*4. Assemblies of the Rcorrector-corrected reads (3 assemblies)*

______________________________________________________________

sim4db2243:sim4begin

2122[425-0-0] 19[149898595-149903020] <425-0-100-complement-unknown>

edef=>Locus_1323_Transcript_1/1_Confidence_1.000_Length_425

ddef=>chr1

1-425 (2001-2425) <425-0-100>

sim4end

sim4db2244:sim4begin

2123[571-0-0] 19[149899037-149903588] <569-0-99-complement-unknown>

edef=>Locus_1324_Transcript_1/1_Confidence_1.000_Length_571

ddef=>chr1

1-571 (1981-2551) <569-0-99>

sim4end

sim4db2242:sim4begin

2121[1868-0-0] 19[149900256-149910518] <1858-0-100-complement-reverse>

edef=>Locus_1322_Transcript_1/1_Confidence_1.000_Length_1868

ddef=>chr1

11-611 (2001-2601) <601-0-100> <-

612-682 (3580-3650) <71-0-100> <-

683-749 (3900-3966) <67-0-100> <-

750-874 (5043-5167) <125-0-100> <-

875-1207 (5247-5579) <333-0-100> <-

1208-1343 (5828-5963) <136-0-100> <-

1344-1422 (6085-6163) <79-0-100> <-

1423-1565 (6623-6765) <143-0-100> <-

1566-1626 (6936-6996) <61-0-100> <-

1627-1748 (7221-7342) <122-0-100> <-

1749-1824 (7791-7866) <76-0-100> <-

1825-1868 (8219-8262) <44-0-100>

sim4end
